# Emerging Evidence of Macrophage Contribution to Hyperinnervation and Nociceptor Sensitization in Vulvodynia

**DOI:** 10.3389/fnmol.2019.00186

**Published:** 2019-08-06

**Authors:** Christine Mary Barry, Dusan Matusica, Rainer Viktor Haberberger

**Affiliations:** ^1^Musculoskeletal Neurobiology Laboratory, Centre for Neuroscience, College of Medicine and Public Health, Flinders University, Adelaide, SA, Australia; ^2^Pain and Pulmonary Neurobiology Laboratory, Centre for Neuroscience, Órama Institute, College of Medicine and Public Health, Flinders University, Adelaide, SA, Australia

**Keywords:** vulvodynia, vestibulodynia, hyperinnervation, nociceptor sensitization, pain, macrophage polarization, nerve growth factor

## Abstract

Vulvodynia is an idiopathic chronic pain disorder and a leading cause of dyspareunia, or pain associated with sexual intercourse, for women. The key pathophysiological features of vulvodynia are vaginal hyperinnervation and nociceptor sensitization. These features have been described consistently by research groups over the past 30 years, but currently there is no first-line recommended treatment that targets this pathophysiology. Instead, psychological interventions, pelvic floor physiotherapy and surgery to remove painful tissue are recommended, as these are the few interventions that have shown some benefit in clinical trials. Recurrence of vulvodynia is frequent, even after vestibulectomy and questions regarding etiology remain. Vestibular biopsies from women with vulvodynia contain increased abundance of immune cells including macrophages as well as increased numbers of nerve fibers. Macrophages have multiple roles in the induction and resolution of inflammation and their function can be broadly described as pro-inflammatory or anti-inflammatory depending on their polarization state. This state is not fixed and can alter rapidly in response to the microenvironment. Essentially, M1, or classically activated macrophages, produce pro-inflammatory cytokines and promote nociceptor sensitization and mechanical allodynia, whereas M2, or alternatively activated macrophages produce anti-inflammatory cytokines and promote functions such as wound healing. Signaling between macrophages and neurons has been shown to promote axonal sprouting and nociceptor sensitization. This mini review considers emerging evidence that macrophages may play a role in nociceptor sensitization and hyperinnervation relevant to vulvodynia and considers the implications for development of new therapeutic strategies.

## Introduction

Vulvodynia is a chronic pain disorder, usually characterized by pain localized to the vaginal entrance (localized provoked vulvodynia; Goldstein et al., 2016). Pain can be intense and may be associated with vaginismus, or spasm of pelvic floor muscles (Goldstein et al., 2016). However, vulvodynia is primarily a pain disorder and not secondary to factors such as vaginismus, disordered arousal or lack of vaginal lubrication (Heim, 2001). Frequently there is no history of trauma or infection, though many women report prior candiasis (Leusink et al., 2018). Women and girls of all ages can be affected but most are in younger age groups and a high proportion under 25 years (Harlow et al., 2014). Many women with vulvodynia are unable to insert a tampon, engage in sexual activity involving vaginal penetration or undergo a gynecological examination. The impact on their self-esteem, relationships and fertility can be substantial.

The key pathological features of vulvodynia are vaginal hyperinnervation (Bohm-Starke et al., 1998; Tympanidis et al., 2003, 2004; Bornstein et al., 2004; Halperin et al., 2005; Goetsch et al., 2010; Leclair et al., 2011; Tommola et al., 2016; Liao et al., 2017) and nociceptor sensitization contributing to mechanical and thermal hyperalgesia (Bohm-Starke et al., 2001). Hyperinnervation includes multiple types of fibers including fibers containing calcitonin gene-related peptide (Bohm-Starke et al., 1999) and fibers expressing the receptor TRPV1 (Bohm-Starke et al., 1999; Tympanidis et al., 2004).

Despite evidence of structural and functional changes related to innervation, the recommended first-line treatments for vulvodynia are psychological interventions, pelvic floor physiotherapy and surgery to remove painful tissue (Goldstein et al., 2016). Whilst these interventions have shown benefit in clinical trials, vulvodynia remains a highly prevalent and recurrent pain disorder (Harlow et al., 2014). To date, clinical trials for treatment targeting the pathophysiology of vulvodynia have not demonstrated benefit (Goldstein et al., 2016). Vestibulectomy is an invasive procedure but an option if conservative treatments fail. Data from large clinical trials are lacking but a relatively recent study found 90% of women reported moderate or substantial improvement (Swanson et al., 2014). Beneficial effects of surgery for vulvodynia, and therefore removal of input from sensitized fibers, indicates peripheral mechanisms make a substantial contribution to the disorder, supporting the view that appropriate interventions targeting peripheral pathology will be beneficial (Keppel Hesselink et al., 2016).

## Immune Cells and Vulvodynia Pathophysiology

In addition to hyperinnervation, vestibular biopsies from women with vulvodynia contain increased abundance of immune cells (Lundqvist et al., 1997; Tommola et al., 2015; Liao et al., 2017) and vaginal swabs contain increased pro-inflammatory cytokines (Zanotta et al., 2018). Increased T cells, B cells and macrophages have been identified in samples from symptomatic areas compared to non-symptomatic areas or healthy controls (Liao et al., 2017). Symptomatic tissue also contained 70% increase in nerve fibers immunoreactive for the pan-neuronal marker PGP9.5, and over 100% increase in the density of TRPV4-immunoreactive, putative mechano-nociceptive fibers (Liao et al., 2017). Increased B lymphocytes, but not T cells or macrophages, have been identified in archival vestibulectomy tissue (Tommola et al., 2015). Conflicting findings are reported regarding the abundance of mast cells in vestibular biopsy samples (Bornstein et al., 2004; Liao et al., 2017). High numbers of immune cells immunoreactive for nerve growth factor (NGF) have been identified associated with intraepithelial nerve fibers in biopsy samples from women with vulvodynia, suggesting NGF may be a pathophysiological factor (Tommola et al., 2016). Fibroblast-mediated pro-inflammatory responses to Candida infection have also been implicated (Falsetta et al., 2015, 2017). Fibroblasts cultured from vestibular tissue of vulvodynia patients showed increased expression of receptors for bradykinin and increased Dectin-1 receptors that bind Candida albicans. These cells showed increased production of pro-inflammatory and proalgesic interleukin (IL)-6 and prostaglandin E2 (PGE2) in response to low-level exposure to Candida albicans or bradykinin stimulation (Falsetta et al., 2015, 2017). Interestingly, patients’ pain sensitivity correlated with levels of cytokines produced by cultured fibroblasts exposed to Candida (Foster et al., 2015), consistent with a localized peripheral pathology making a substantial contribution to patients’ pain.

Macrophages have an established role in many conditions associated with chronic pain (Pinho-Ribeiro et al., 2017) but few studies have investigated macrophage-neuron interactions that may contribute to hyperinnervation and nociceptor sensitization in vulvodynia. Macrophages are a heterogeneous population of cells with multiple functions in development, homeostasis and disease. In addition to phagocytosis of foreign pathogens and apoptotic cells, macrophages release hundreds of effector molecules and proteins including growth factors, cytokines and chemokines (Mantovani et al., 2005). They have high plasticity and depending on their phenotype or polarization state, make contributions to both the induction and resolution of inflammation. According to a simplified descriptive framework, M1, or classically activated macrophages promote inflammation and hyperalgesia. Their major release factors include reactive oxygen and nitrogen species, and the pro-inflammatory cytokines IL-1α, IL-1β, TNFα and IL-6 (Mantovani et al., 2005; Liu et al., 2019). M2 or alternatively activated macrophages have anti-inflammatory effects and promote hypoalgesia (Leung et al., 2016; Pannell et al., 2016; Huo et al., 2018). In addition to releasing IL-10, subtypes of M2 macrophages (M2a, M2b, M2c and M2d), contribute to functions that promote cell proliferation, cell maturation, resolution of inflammation and angiogenesis (Liu et al., 2019). The clear differentiation between M1 and M2 macrophages that can be seen *in vitro* does not fully represent the complex array of functional and phenotypic states found *in vivo*, including many transitional states of activation finely tuned to different microenvironments and also dependent on tissue specificity (Gordon and Taylor, 2005; Mosser and Edwards, 2008; Cassetta et al., 2011; Lawrence and Natoli, 2011; Murray and Stow, 2014). Distinguishing macrophage subsets based on the distinct expression of surface markers remains a challenge and overlap of antigenicity of subtypes is substantial, but a growing number of studies apply the M1/M2 framework to compare macrophage abundance and activation state in injury or disease conditions and in response to interventions.

In homeostatic conditions, a heterogeneous population of macrophages maintains a state of dynamic equilibrium within tissue, and those including embryonically derived tissue-resident macrophages derived from circulating monocytes of bone marrow origin (Jenkins et al., 2011; Epelman et al., 2014). In many tissues such as liver and skeletal muscle, influx of circulating monocytes and their differentiation into M1 macrophages is a critical part of the acute inflammatory response (Duffield et al., 2005; Arnold et al., 2007). Within tissue, both recruited and resident macrophages have the capacity for proliferation (Epelman et al., 2014). In response to injury, macrophages of different phenotypes are present simultaneously and work synergistically (Duffield et al., 2005). M1 macrophages transition to M2 phenotypes in a cytokine-driven process critical for repair and remodeling (Arnold et al., 2007; Dal-Secco et al., 2015). For example, in injured skeletal muscle, transition of M1 (CX3CR1^lo^/Ly6C+) macrophages to M2 (CX3CR1^high^/Ly6C-) phenotype has been shown following phagocytosis of muscle cell debris, and whereas M1 macrophages coculture promoted proliferation of muscles cells, coculture with M2 macrophages stimulated cell growth. The critical role of macrophages is highlighted by observations that macrophage depletion at the time of injury prevents muscle repair (Arnold et al., 2007).

## Macrophages and Regulation of Nociceptive Signaling

Macrophages are implicated in the regulation of pain sensitivity in multiple conditions (Gong et al., 2016; Shepherd et al., 2018; Sakurai et al., 2019). Macrophage infiltration has been demonstrated in pain-associated synovial tissue from patients with advanced osteoarthritis and in pain-associated models of joint, muscle and paw inflammation (Gong et al., 2016; Shepherd et al., 2018; Sakurai et al., 2019). More importantly, macrophage depletion *via* clodronate liposomes reduces the elevated pro-inflammatory cytokines and NGF and reduces pain behaviors in a model of arthritis (Sakurai et al., 2019). Similarly, macrophage depletion prevents local hyperalgesia in response to plantar injection of angiotensin II (Shepherd et al., 2018), and widespread hyperalgesia in response to repeated intra-muscular injection of acidic saline and pro-inflammatory agents (Gong et al., 2016). Macrophage blockade using a toll-like receptor 4 antagonist also prevents hyperalgesia in this model (Gong et al., 2016). Increased abundance of ED-1+ monocytes/macrophages in injured rat sciatic nerves correlates with allodynia (Cui et al., 2000) whereas macrophage depletion alleviates thermal hyperalgesia following rat sciatic nerve ligation (Liu et al., 2000) and prevents mechanical allodynia associated with chemotherapy-induced peripheral neuropathy (Sekiguchi et al., 2018). Macrophage to neuron signaling, particularly nociceptor sensitization *via* the release of proalgesic cytokines, is well established.

Apart from animal models, there has been increased interest in identifying macrophage phenotypes in conditions associated with chronic pain in humans. Synovial fluid from patients with knee osteoarthritis was found to contain markedly higher ratios of M1 (CD11c+) to M2 (CD206+) macrophages compared to healthy controls, and this ratio correlated with measures of radiographic joint disease (Liu et al., 2018). Cadaveric intervertebral discs with degenerative changes contained increased M1 (CCR7+) macrophages and subtypes of M2 macrophages (M2c, CD163+), specifically localized in areas of nucleus, annulus and vertebral endplate showing structural defects (Nakazawa et al., 2018). These findings are supported by a mouse model of intervertebral disc injury that showed increased M1 macrophages at day 1 returning to normal levels at 28 days, and increased M2a (CD206+) and M2c macrophages (CD163) at days 7, 14 and 28 (Nakazawa et al., 2018).

The shift from M1 to M2 phenotypes appears critical for resolution of protective hyperalgesia associated with the acute inflammatory response. Spinal cord injury, a condition frequently associated with prolonged neuropathic pain, results in a sustained increased abundance of M1 phenotype cells in the spinal cord (Kigerl et al., 2009; Pruss et al., 2011). These cells produce pro-inflammatory and cytotoxic cytokines and they include M1 macrophages derived from circulating monocytes and activated microglia sharing the same antigenicity and morphology (David and Kroner, 2011). In rat models of spinal cord injury, a relatively brief increase in the total number of M2 (CD206+) macrophages/microglia is accompanied by a greater, sustained accumulation of M1 (arginase+) macrophages, with high ratios of M1 to M2 polarized macrophages at 28 days (Kigerl et al., 2009) and 70 days following injury (Pruss et al., 2011). This has implications for pain sensitivity as well as secondary injury such as demyelination. Bone cancer pain is another clinical challenge in which altered macrophage phenotype has been implicated. In a mouse model of bone cancer pain, increased M1 (iNOS+, CD16/32/Iba1+) spinal cord macrophages/microglia were identified, with increased production of IL-1β and reduced production of IL-10 (Huo et al., 2018). Administration of dehydrocorydaline, an alkaloidal component isolated from Rhizoma corydalis previously shown to reduce inflammatory pain (Yin et al., 2016), resulted in increased M2 (CD206/Iba1) spinal cord microglia/macrophages and reduced pain behavior (Huo et al., 2018).

Since vulvodynia is a condition affecting women, sex-related differences regarding nerve-immune cell interactions regulating nociceptive signaling are important to consider. Sex differences in clinical pain are well established, including higher prevalence of chronic pain among women compared to men, greater sensitivity of women to multiple measures of experimentally induced pain and different analgesic responses to opioid drugs (Bartley and Fillingim, 2013; Sorge and Totsch, 2017). Multi-disciplinary studies have implicated multiple biological, psychological and social factors. Interactions between sex hormones and the immune system, particularly involving microglia and T cells, are proposed to account for much of the biological component (Sorge and Totsch, 2017). Less is documented regarding sex-related differences in macrophage regulation of nociception, though ovarian hormone regulation of macrophage phenotype and number (Scotland et al., 2011) may show macrophages are particularly important for pain in females.

Microglial promotion of spinal cord neuron hyperexcitability in models of neuropathic and inflammatory pain appears more important in males than females. Intrathecal LPS to activate microglia produces allodynia only in male mice (Sorge et al., 2011) and analgesic responses to microglial inhibitors are testosterone dependent (Sorge et al., 2015). T cells may also contribute to sex-related differences in pain. T cells are more abundant in female compared to male mice and, partly due to diminished testosterone-related inhibition, produce more pro-inflammatory, proalgesic mediators (Sorge et al., 2015). Whereas female CD-1 mice require 2–3-fold more morphine for an equivalent analgesic response to males, the difference is abolished in T-cell deficient mice (Rosen et al., 2019). Female rodents have greater abundance of macrophages in peritoneal and pleural cavities, higher TLR expression by resident macrophages and greater production of pro-inflammatory cytokines on macrophage stimulation (Scotland et al., 2011; Ćuruvija et al., 2017). These findings support the view that further investigation into the role of macrophages in chronic pain conditions affecting women is warranted.

In summary, data from human studies and animal studies investigating chronic inflammatory, neuropathic and cancer pain, all support the view that macrophage phenotype may be an important peripheral factor influencing pain sensitivity. Additionally, there is evidence M2 macrophages promote analgesia. In a model of peripheral neuropathic pain, application of cultured M2 macrophages to the injured nerve reduced mechanical pain sensitivity (Pannell et al., 2016), possibly due M2 macrophage production of endogenous opioids (Pannell et al., 2016). Interestingly, regular exercise promotes M2 macrophage polarization in skeletal muscle (Leung et al., 2016) and protects against hyperalgesia in models of chronic muscle and paw pain, with no sex-related differences identified (Leung et al., 2016).

## Macrophage-Neuron Signaling and Hyperinnervation

Macrophage to neuron signaling may promote axonal sprouting and hyperinnervation. Pronounced and persistent hyperinnervation has been described following inflammation in skin (Reynolds and Fitzgerald, 1995; Chakrabarty et al., 2013), synovium (Ghilardi et al., 2012), muscle (Ambalavanar et al., 2006) and deep fascia (Hoheisel et al., 2015), and in association with painful endometriotic lesions (Anaf et al., 2000; Morotti et al., 2014). This hyperinnervation involves sensory A- and C-fibers and is accompanied by mechanical and thermal hypersensitivity (Reynolds and Fitzgerald, 1995; Chakrabarty et al., 2013). All of these tissues contain abundant macrophages that release effector molecules and growth factors shown to promote hyperinnervation. Few studies have investigated the specific contributions of macrophages to hyperinnervation, though macrophages are shown to regulate the regeneration of injured peripheral nerves, by sensing hypoxia at the nerve bridge, recruiting endothelial cells and driving neovascularization critical for Schwann cell migration (Cattin et al., 2015). *In vitro* studies show distinct effects of M1 and M2 macrophages on neuronal growth and survival (Kigerl et al., 2009). Adult DRG neurons incubated in M1 macrophage conditioned media show shorter, more highly branched neurites whereas those incubated in M2 conditioned media showed a uni- or bi-polar phenotype with elongated axons. M1 conditioned media was toxic to cortical neurons whereas M2 conditioned media was not (Kigerl et al., 2009).

Cutaneous hyperinnervation induced by plantar injection of complete Freund’s adjuvant is accompanied by abundant angiotensinogen and renin production in macrophages and T cells (Chakrabarty et al., 2013). Hyperinnervation and hyperalgesia were prevented by an angiotensin receptor II antagonist, indicating angiotensin II produced by macrophages and T cells modulates sensory fiber sprouting. Subsequent research by this team found vestibular biopsies from women with vulvodynia contain increased macrophages and T cells expressing renin and angiotensinogen (Liao et al., 2017), and that an angiotensin receptor II antagonist prevented vaginal hyperinnervation in rats in response to CFA (Chakrabarty et al., 2018).

The ability of macrophages to induce nerve sprouting is shown in sympathetic hyperinnervation following myocardial infarction in a mechanism involving their production of NGF (Hasan et al., 2006; Wernli et al., 2009). NGF also contributes to sensory hyperinnervation and hyperalgesia in response to inflammation (Woolf et al., 1994), and anti-NGF therapy has anti-nociceptive effects in the treatment of arthritis (Sakurai et al., 2019). Macrophages are an important source of NGF following injury (Lindholm et al., 1987). NGF not only acts on nerve fibers, but also acts on macrophages, potentially affecting polarization state. In cultured macrophages, NGF promotes cell survival and alters the release of 53 of 507 proteins secreted by macrophages, including growth factors, cytokines, and chemokines (Williams et al., 2015). Regarding proteins associated with classically activated or alternatively activated macrophages, NGF stimulation increased macrophage secretion of TGF-β and decreased secretion of IL-10, IL-1α and IL-1β (Williams et al., 2015).

Injury models indicate that actions of NGF mediated by macrophages may be sustained for prolonged periods. In the intervertebral disc injury model, injured discs contain increased abundance of macrophages, NGF mRNA and NGF protein 1 day following injury, and all three measures continue to be substantially increased 28 days following injury (Nakawaki et al., 2019).

## Summary and Conclusions

For many years lack of suitable models of vulvodynia was a major barrier to the development of treatments that specifically target the pathophysiology of the disease. Recently developed models of vaginal hyperinnervation in rats and mice are an important advancement (Farmer et al., 2011; Barry et al., 2018; Chakrabarty et al., 2018; Sharma et al., 2018). Increased abundance of macrophages has been observed in these models accompanying increased vaginal nerve fibers, consistent with signs in patient biopsies, but the extent to which macrophages contribute to hyperinnervation or nociceptor sensitivity in vulvodynia remains unclear. Indeed, macrophage polarization state has not yet been described in clinical vulvodynia or in models, and the impact of macrophage depletion has not been identified. Therefore, direct evidence for a specific role of macrophages in vulvodynia is lacking.

However, a significant body of research demonstrates macrophages can contribute to hyperinnervation and nociceptor sensitization in multiple pathological conditions. Therapeutic approaches that target angiotensin signaling, putatively involving macrophages, appears promising in addressing key pathological features of vulvodynia. As with other organs in the body, the composition of embryonically and adult-derived macrophage subpopulations in the vagina is not yet clear, nor the extent to which local proliferation and circulating monocytes replenish and expand populations within a tissue in homeostatic and disease states. This could have implications on the effectiveness of interventions targeting monocyte migration or proliferation of subtypes of macrophages, in addition to factors altering macrophage polarization state. Studies clearly show macrophages are highly dynamic and can transition between polarization states that have distinct effects on nociception, suggesting they are a potential target for interventions to modulate pain sensitivity. Modulation of the microenvironment by interventions including exercise, can alter macrophage phenotype and shift the balance of their functions and potentially protect against the development of chronic pain.

## Author Contributions

CB designed and drafted the manuscript. CB, DM and RH contributed to manuscript critical revision and read and approved the submitted version.

## Conflict of Interest Statement

The authors declare that the research was conducted in the absence of any commercial or financial relationships that could be construed as a potential conflict of interest.
